# A league of their own: demographics, motivations and patterns of use of 1,955 male adult non-medical anabolic steroid users in the United States

**DOI:** 10.1186/1550-2783-4-12

**Published:** 2007-10-11

**Authors:** Jason Cohen, Rick Collins, Jack Darkes, Daniel Gwartney

**Affiliations:** 1 Arlington, VA, USA; 2 Collins, McDonald & Gann, P.C., Carle Place, NY, USA; 3 Department of Psychology, University of South Florida, Tampa, FL, USA; 4 Alcohol and Substance Abuse Institute, University of South Florida, Tampa, FL, USA; 5 Columbia, MO, USA

## Abstract

**Background:**

Rule violations among elite-level sports competitors and tragedies among adolescents have largely defined the issue of non-medical anabolic-androgenic steroid (NMAAS) use for the public and policy makers. However, the predominant and oft-ignored segment of the NMAAS community exists in the general population that is neither participating in competitive sports nor adolescent. A clearer profile of NMAAS users within the general population is an initial step in developing a full understanding of NMAAS use and devising appropriate policy and interventions. This survey sought to provide a more comprehensive profile of NMAAS users by accessing a large sample of user respondents from around the United States.

**Methods:**

U.S.-based male NMAAS users (*n *= 1955) were recruited from various Internet websites dedicated to resistance training activities and use of ergogenic substances, mass emails, and print media to participate in a 291-item web-based survey. The Internet was utilized to provide a large and geographically diverse sample with the greatest degree of anonymity to facilitate participation.

**Results:**

The majority of respondents did not initiate AAS use during adolescence and their NMAAS use was not motivated by athletics. The typical user was a Caucasian, highly-educated, gainfully employed professional approximately 30 years of age, who was earning an above-average income, was not active in organized sports, and whose use was motivated by increases in skeletal muscle mass, strength, and physical attractiveness. These findings question commonly held views of the typical NMAAS user and the associated underlying motivations.

**Conclusion:**

The focus on "cheating" athletes and at risk youth has led to ineffective policy as it relates to the predominant group of NMAAS users. Effective policy, prevention or intervention should address the target population(s) and their reasons for use while utilizing their desire for responsible use and education.

## Background

As many as 3 million Americans may have used anabolic-androgenic steroids (AAS) for non-medical purposes [1]. However, concerns over non-medical AAS (NMAAS) use have been motivated less by prevalence in the general population than by NMAAS in two specific subpopulations: athletes contravening the rules of elite-level sports [2-5] and minors [6,7]. Such concerns essentially dominated the media and policy debate when AAS control legislation was enacted in 1990 and amended in 2004. In a time marked by global terrorism and potential ecological crises, the President of the United States stated during the 2004 State of the Union address to note that the "...use of performance-enhancing drugs like steroids in baseball, football and other sports is dangerous, and it sends the wrong message – that there are shortcuts to accomplishment, and that performance is more important than character" [8].

Detailed information on NMAAS and its motivations are difficult to obtain due to the legal implications and the subsequent wariness within the NMAAS subculture [9,10]. Most prevalence estimates of use emerge from larger surveys of drug use among high school and college students [7,11-18] and are fielded periodically in school settings [13,19], surveying large national samples. However, such surveys often collect only limited information on NMAAS use, such as lifetime, past year, and past month use with no data indicating the rate of repeated use of AAS among adolescents. This focus on secondary and collegiate students partly reflects concerns for the profound effects of substance use during adolescence [20] as well as concerns for recent rare and tragic teenage suicides that were possibly associated with mismanaged cessation of NMAAS use [21,22].

In the case of NMAAS use among elite athletes, although highly visible and widely publicized, it is almost certain that the attention garnered exaggerates the contribution to overall prevalence of NMAAS use; such athletes likely comprise only a minor percentage of the NMAAS using population [7,23-25]. In fact, researchers claim that "The large majority of anabolic steroid users are not elite athletes" [8].

Though prevalence rates derived from surveys in educational settings or discussion of elite athlete use provide useful information on use patterns and trends over time in certain populations, they tell us nothing about the characteristics of those who self-administer AAS for non-medical purposes. In fact, despite calls for a more complete characterization of NMAAS users more than 15 years ago [26], questions still remain: Who among the general population are using AAS? Why and how do they use them? When did they begin using them? Most of what is known about the onset and patterns of, and motivations for, NMAAS use has been derived from small, non-representative samples of users [27-29], or case reports [30]. Such small selective samples from limited geographical areas are not likely to accurately characterize the general NMAAS-using population. Therefore, this survey sought to provide a more complete profile of NMAAS users by accessing a large sample of user respondents from around the United States via various Internet websites and magazines dedicated to resistance training activities and use of ergogenic substances. It is hoped that the resulting information on NMAAS use – who, what, why, when and how – would increase understanding of those who self-administer NMAAS and thereby increase understanding relevant to social policy, risk identification, prevention, and treatment.

## Methods

### Recruitment strategy

The illicit nature of NMAAS use can hamper traditional recruitment efforts. Users often have justifiable concerns about confidentially when responding to questionnaires in person or by mail. Conversely, the resources required to personally interview a large representative sample of participants can be prohibitive. Thus, most large scale surveys focus solely on prevalence and most in-depth studies use either small local samples or select groups (e.g., prisoners or patients in treatment).

To circumvent those concerns, promote participation, and facilitate recruitment, an Internet-based survey tool was designed. The Internet has become the primary means of buying and selling illicit AAS [31] and a primary source of NMAAS information [32]. Most NMAAS users are likely to be experienced with the Internet and its use in NMAAS-related activity. This approach allowed for anonymity and enhanced privacy and confidentiality, and also facilitated access to a wide range of geographical areas. It has previously been used in NMAAS surveys [33,24,32]. Web-based surveys provide a validated method for collecting self-reports of substance use [34-36] and efficient access to large representative samples of specialized groups [37]. Further, their validity has been supported by their consistency with other data collection methods [38,39].

A written request for participation, including a brief explanation of the purpose and scope of the survey, emphasizing participants' privacy and the researchers' objectivity and interest in participants' "candor," "honesty" and "truthfulness", was posted to several venues.

### Recruitment methods

1) *Internet posts *– A URL link to the web-based survey was posted on 12 online message boards where steroid discussion is commonplace. The message boards attract a broad range of individuals to discuss topics such as bodybuilding, strength, fitness, diet, nutritional supplements, sports, and NMAAS use. A link was also placed on an educational site [40] operated by one of the authors (R.C.) These materials are known to have migrated (see # 4 below), from their original sites, although the full extent of migration is unknown.

2) *Mass emails *– Three of the above-referenced message boards sent an email requesting participation to all registered users.

3) *Print media *– A brief description of the survey, including the URL, was printed in a popular bodybuilding magazine (*Muscular Development*, 12/05).

4) *Spontaneous network recruiting *– Participants, on their own (without solicitation), passed information about the survey's existence to others.

The survey was fielded for four months. Only those with Internet access who chose to participate after reading about the study were included. No data is available to compare participants to NMAAS users without Internet access, those unaware of the survey, or those who chose not to participate.

### Instrumentation

Clicking the URL opened an informed consent page constructed in accord with the American Psychological Association (APA) Ethical Principles of Psychologists and Code of Conduct [41]. Privacy and confidentiality were insured in several ways: No identifying data were collected. Internet Provider (IP) addresses were not logged, so responses could not be linked to a specific computer. Secure Sockets Layer (SSL) 128 bit encryption and 1024 bit exchange facilitated secure transfer of data. Data were secured in an encrypted, password-protected hidden vault on a dedicated computer. An Internet cookie placed on respondents' machines allowed completion of the survey over multiple sessions if desired and discouraged multiple submissions. Respondents were informed about the cookie and, upon starting and completing the survey, provided instructions deleting it. The survey blocked any respondent who did not consent, indicated they were less than 18 years old, did not use AAS for non-medical purposes, or had previously taken the survey.

The survey included 291 items assessing various domains, including demographic/background data, AAS use patterns and purchasing behavior, positive and negative physiological and psychological side effects, health and mental health history, other drug use, and dietary practices. A subset of the data is presented herein to describe the users of AAS, their motivations, history, methods and practices of use.

Respondents rated the effectiveness while considering side effects of a variety of AAS and other drugs on a 5-point likert-type scale from 1 (very poor) to 5 (very good) in response to the following statement: "After considering side effects, please rate the following in how effective and useful they are in helping you reach your goals". Respondents who had not used an agent that was to be rated were requested to skip that item. The effectiveness of ancillary drugs were rated on 3-point likert-type scale (1 [not effective], 2 [moderately effective], 3 [highly effective]) or a box indicating they had never used the agent. NMAAS use motivations were rated on a 5-point likert-type scale from 1 (not a reason for use) to 5 (very important) in response to the stem "How much do the following items (15) motivate your use?" The survey software randomized the order of presentation. Concerns for aversive effects upon cessation as motivation (negative reinforcement) were assessed via endorsement of the following outcomes should access to AAS be lost or AAS use ceased: "Nothing, this would not be an issue for me", "Losing size/getting small", "Losing strength", "Losing respect", "Being unattractive", "Decreased ability to compete in sports" and "Other" which allowed an open-ended response. Sports involvement at the high school, college, amateur, Olympic and professional levels, as well as occupation and age, were obtained via open-ended questions. Dietary regimen questions were rated on a 5-point likert-type scale.

Past behavior (e.g., age of onset of AAS use, high school athletic activities) was also assessed. Although such queries can be subject to hindsight bias, participants are normally able to reliably provide valid historical information [42] and AAS users especially have "...an uncanny ability..." to recall their AAS use history from as many as 20 years earlier [10].

To enhance motivation and attention, skip logic was employed; participants responded only to personally-relevant items based on prior responses. For this reason, not all participants answered all items and, therefore, the number of responses varied from domain to domain. In addition, not all participants responded to all relevant items (such sporadic missing data is not uncommon in large surveys; [43]). Hence, proportions of participants responding to items of interest are reported. The survey took 30–45 minutes to complete.

### Data analysis

SPSS for Windows (version 13) was used for statistical analyses. Descriptive statistics (e.g., means, medians, modes, ranges and standard deviations) are provided where applicable and in certain areas, descriptive comparisons are made with U.S. Census data. Medians were reported rather then means when data were skewed. Scale means, based on the 5-point likert-type format noted above are presented in some areas. Pearson's product movement correlations (*r*) evaluated relationships between interval data.

## Results

The full sample comprised 2,663 males and females from 81 countries. To control for gender and cultural differences in NMAAS use and national differences in the legal status of AAS, this report focuses only on NMAAS use among American males. The final analysis sample in the current report included 1,955 American males engaged in NMAAS use.

### Who is using AAS?

#### Age and marital status

**Figure 1:**
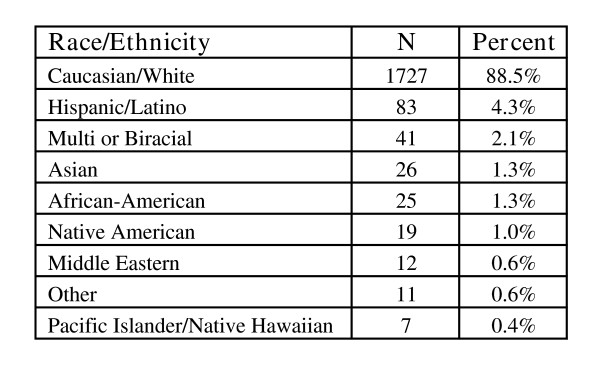
Race/Ethnicity.

The average AAS user was 31.1 years of age (*SD *= 9.16; age range = 18 – 76) and the median age was 29 years. An overwhelming majority (88.5%) were Caucasian/White (see Figure 1). About half had never been married (51.38%), although many were currently married (38.38%) and some were divorced (9.09%). Consistent with the largely unwed status of the sample, most did not have children (64.21%).

#### Education, employment and income

**Figure 2:**
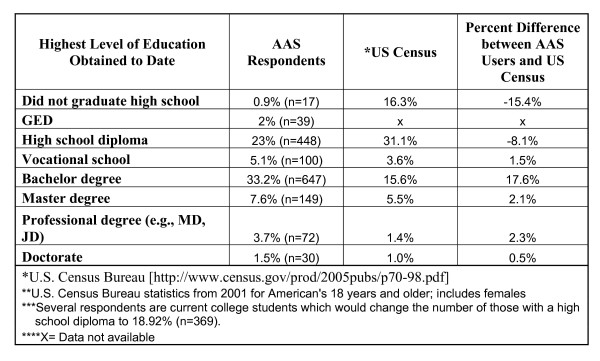
Level of Education.

**Figure 3:**
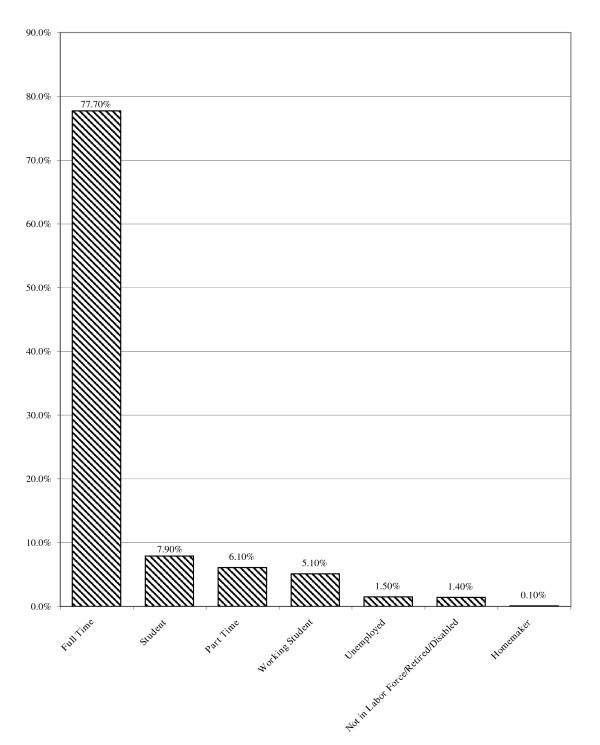
Employment Status.

**Figure 4:**
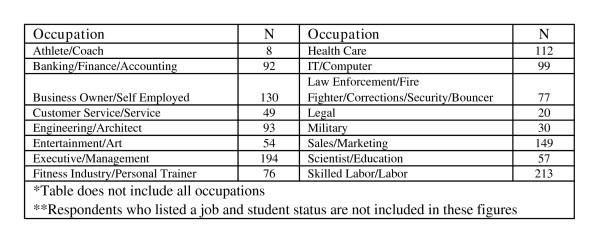
Occupations.

**Figure 5:**
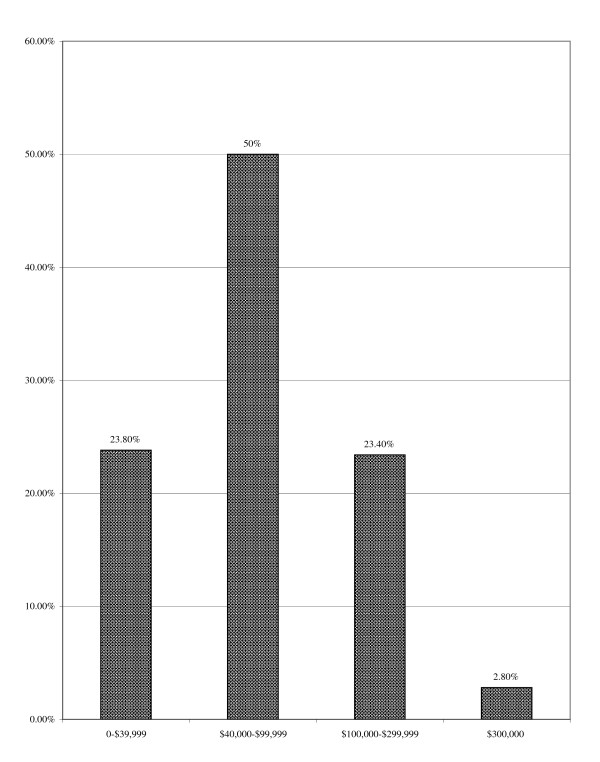
Annual Household Income.

The group was well-educated; most held post-secondary degrees (74.1%) and, compared to recent U.S. Census statistics, more had completed college and advanced degrees and fewer had failed to graduate high school than expected based on the general populace (see Figure 2). Most were employed full-time (77.7%; see Figure 3) and the overall employment rate of 98.5% was higher than for males aged 20 years or more in the U.S. population (72.4% as of November, 2005; [44]). The unemployment rate for males aged 20 years and older in the U.S. in November, 2005 was 4.3% [44], nearly three times the 1.5% unemployment rate observed among this NMAAS-using sample. Most were employed as professionals (i.e., "white collar" employees; see Figure 4) with median household income between $60,000 and $79,999 per year, much higher than the general population ($44,684[45]; see Figure 5). Such above-average educational and occupational functioning appear consistently among AAS users (see also [25]).

#### Users' perceptions

Compared to others, respondents considered their drive and motivation in the "average/above average to above average" range. Most responded as setting "average/high to high" goals and a majority (70.2%) self-identified as "perfectionists". They tended to view "some to some/all" of life as a competition and felt that "half to most (75%)" of daily activities focused on goal achievement.

In sum, NMAAS use was associated with a relatively high level of functioning. Users self-identified as being driven and motivated, viewed life competitively, and focused on goal achievement. It must be noted, however, that, although Internet surveys are a validated methodology and 70% of Americans (82% of those between the ages of 18 and 49) use the Internet [46], the possibility that the use of an Internet survey strategy could have lead to an over-sampling of those with higher education and socio-economic status cannot be completely ruled out.

### What agents are being used and how are they obtained?

#### Popularity of various AAS agents

**Figure 6:**
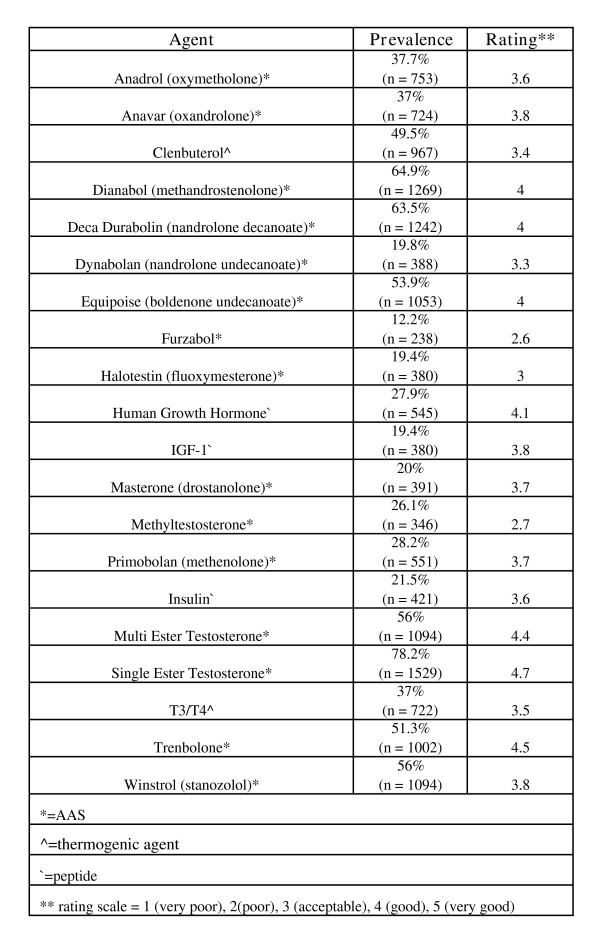
Prevalence and Ratings for Various Agents.

**Figure 7:**
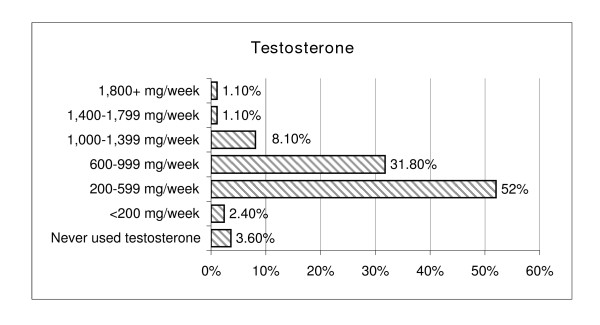
Typical Weekly Testosterone Dosage.

**Figure 8:**
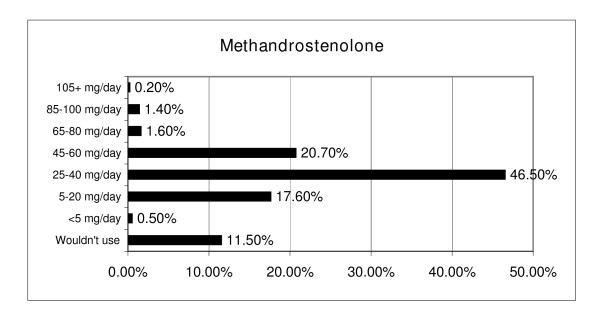
Typical Daily Methandrostenolone Dosage.

Reports of use and effectiveness ratings while considering side effects were obtained for 15 AAS agents. Single ester testosterones, methandrostenolone, and nandrolone decanoate were the most commonly used agents and single and multi-ester testosterones and trenbolone were rated most effective/useful (see Figure 6. Average total AAS dosages ranged from <200 mg (n = 59, 3.6%) to more then 5,000 mg/week (n = 2, 0.1%) with an average of 500–1000 mg/week. The highest dosage of testosterone used for four or more weeks had considerable variability with an average dosage of 797.5 mg/week (sd = 540.11, range = <200 to 10,000 mg/week). Typical weekly testosterone and methandrostenolone dosages are listed in Figures 7 and 8 respectively.

**Figure 9:**
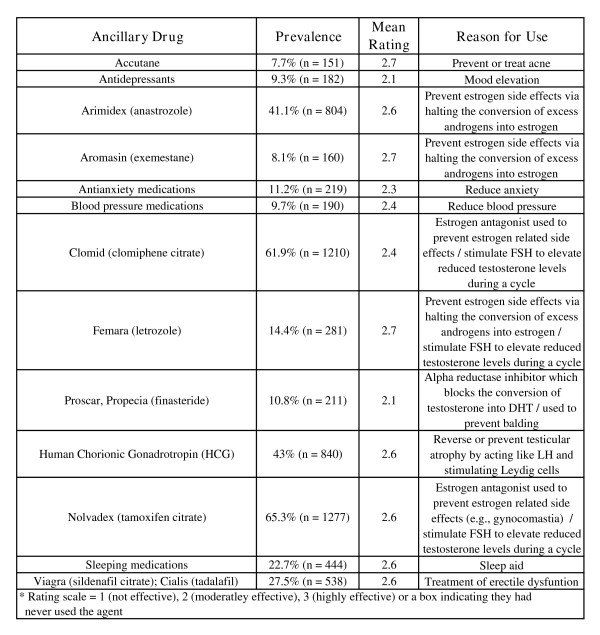
Ancillary Drugs.

NMAAS users also make use of thermogenic agents. These agents are primarily used to reduce body fat with some providing the additional ergogenic benefit of beta-adrenergic stimulation (see Figure 6). NMAAS users have also complemented the ergogenic pharmacopeia to include peptide hormones (e.g., human growth hormone (HGH), insulin-like growth factor (IGF-1), insulin; see Figure 6). Ancillary drugs are also used by NMAAS users to prevent or treat side effects or increase the effectiveness of AAS (see Figure 9).

#### Methods of obtaining AAS

Consistent with the Internet having become a major source for obtaining AAS, half of our sample (52.7%) had purchased AAS over the Internet. Smaller percentages obtained AAS via local sources (16.7%), friends or training partners (15%), physician's prescription (6.6%), or transporting them from foreign countries (5.8%). Some participants reported using multiple methods for procurement and others (0.92%, n = 18), in keeping with privacy/confidentiality concerns, were reluctant to provide this information.

### Why are AAS being used?

#### Positive motivations/reasons for AAS use

**Figure 10:**
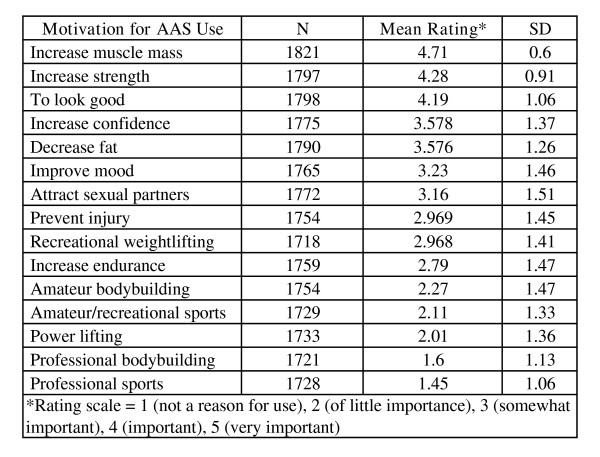
Motivation for AAS Use.

The most highly-rated motivations were increased muscle mass, increased strength and enhanced physical appearance (see Figure 10). Other relevant but less highly-rated factors included increased confidence, decreased fat, improved mood and attraction of sexual partners. Injury prevention, recreational weightlifting, increased endurance, amateur bodybuilding, amateur/recreational sports and power lifting were rarely endorsed motives. AAS' psychotropic effects have been posited as a means whereby AAS dependence might occur [47]; however, virtually all users in our sample (98.8%) denied injecting AAS in order to get "high."

#### Athletics as a motivator

The literature suggests that NMAAS use is rarely, in a statistical sense, motivated by sports participation. Our data showed this as well; 85.1% and 89.2% of NMAAS users, respectively, reported that professional bodybuilding and professional sports did not motivate their NMAAS use, making these the least motivating factors. Only 6.3% and 5.8% respectively indicated bodybuilding and professional sports were "very important" factors in their desire to use AAS.

**Figure 11:**
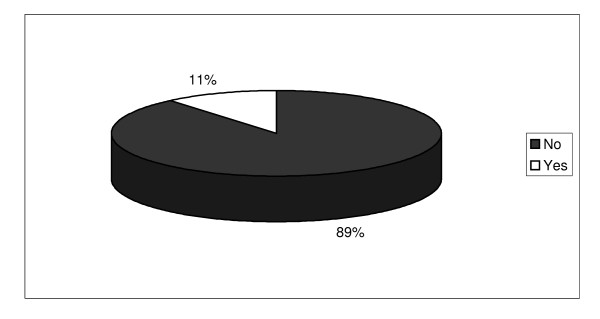
Percent of Respondents who are Current Athletes.

Involvement in any sport, including high school, college, amateur, Olympic or professional sports was rare; most were not involved in organized sport (89%) even when non-traditional sports, such as mixed martial arts, and recreational activities, such as amateur baseball, were included (see Figure 11). At the most common level of organized sports, high school athletics, 81.8% of current users had not participated in high school sport [s]. A minority (4.1%) had played a high school sport and used AAS prior to age 18, although data on the concurrence of these behaviors was not available. Although, as with athletics, bodybuilding is often seen as a major motivation for NMAAS, 84.34% had never competed in any bodybuilding contest, while 15.54% competed as amateurs and only 0.10% had competed professionally in bodybuilding.

#### Negative reinforcement/reasons to continue NMAAS

Complementary to the positive reinforcement motivations endorsed, when asked about aversive factors motivating continued use (i.e., concerns over cessation), loss of muscle mass was the most frequent concern (37%), followed by strength loss (27.2%), decreased attractiveness (12.4%), decreased physical ability (7.2%) and loss of respect (6%). Notably, 30.6% considered the possible loss of access to AAS a non-issue.

#### Effects of age and life stage on motivation for NMAAS

Increasing age within the sample was associated with decreases in several motivations for NMAAS; professional bodybuilding [*r*(1707) = -.126; *p *= .001], attracting sexual partners [*r*(1754) = -.105; *p *= .001], increasing muscle mass [*r*(1801) = -.103; *p *= .001], professional sports [*r*(1712) = -.097; *p *= .001], preventing injury [*r*(1738) = -.094; *p *= .001], recreational weightlifting [*r*(1703)= -.090; *p *= .001], amateur/recreational sports [*r*(1714) = -.088; *p *= .001], increasing strength [*r*(1708) = -.078; *p *= .001], and increasing confidence [*r*(1758) = -.061; *p *= .010]. Conversely, older AAS users were more motivated by decreasing fat [*r*(1771) = .124, *p *= .001]. Most of these changes, such as age-related decreases in a desire for increased muscle, strength, and sexual attraction and increased interest in fat reduction appear to reflect expected shifts in focus based on development. Improving mood, appearance, endurance, power lifting and amateur bodybuilding were not correlated with age.

### When are AAS being used?

#### Age of initial NMAAS and use history

Estimates for 2005 suggested that 2.6% of 12th graders had used AAS in their lifetime, down from a high of 4.0% in 2002 [19]. This study addresses a slightly different question: What is the average age of initiation and the prevalence of adolescent NMAAS use onset among adults who are currently using AAS? That is, do most adult users initiate NMAAS as adolescents?

The average age of NMAAS use onset was 25.81 years old (*sd *= 8.26), agreeing with other reports of NMAAS use onset in the mid-20s [25,48-51]. The youngest reported age of onset was 14 years (*n *= 1) and the oldest was 68 years (*n *= 1). Initiation of NMAAS use was almost exclusively an adult phenomenon; 94% commenced use at age 18 or older. The average user had used AAS, from onset to the present, for 5.53 (*sd *= 5.92) years, ranging from less than 1 year to 43 years of cycling of NMAAS. Most (61.0%) initiated NMAAS within the first five years of weight training.

### How are AAS being used?

#### Training experience and practices

Users averaged 11.07 years (SD = 6.21) of weight training and the majority (69.6%) averaged four to five workout days per week. Most maintained a fairly standard training regimen and few (0.90%) trained seven days per week, a level at which some concern might be noted.

#### Dietary regimen

For a large majority (88.4%), the preponderance (75%) of their daily diet "always" or "frequently" included lean protein consumption and almost half (46.5%) reported consuming "a lot more" than 6 to 10 servings of protein-based foods on a daily basis. Fried food was largely "always" or "frequently" limited (71.3%) and consumed less than once per week (77.6%); three-quarters (76.2%) limited saturated fat intake. Most (73.2%) consumed "a lot less" than one sugar-containing soft drink daily, with many (41%) restricting carbohydrates to "a lot less" or "a little less" than seven servings per week. More than one-quarter (26.4%) reported consuming about 3 to 5 servings of milk daily, with an additional 44.7% consuming "a little" or "a lot more" and 28.9% consuming "a lot" or "a little less". The majority reported consumption of less than 3 to 5 servings of fruit (62%) or vegetables (48.1%) daily.

#### Cycling of NMAAS

AAS are typically cycled, with periods of use interspersed with periods of recovery/abstinence, to allow the endocrine systems of the body to return to homeostasis. There was considerable variability in cycle length (range of 1 week [n = 1] to 728 weeks [n = 1]), with a median of 11 weeks. Most had administered AAS for a total of 5 of the preceding 12 months; 13.5% had not used AAS during the past year and 5% had used AAS for the entire previous year. The average year included 4 to 6 months of use; however several (16.8%) did not answer or could not provide an estimate due to variability in their cycling history. The modal longest on-cycle period was 12 weeks.

#### Cycle planning and preparation

Most ("75–100%") AAS needed for a cycle were obtained prior to beginning a cycle by most users (80%). Ninety-six percent planned the length, dosages and compounds prior to beginning a cycle; 2/3 (69.3%) "always" kept to their predetermined plan and an additional 30.6% "frequently" did so. Cycles were altered to increase (18.7%,) or decrease (13%) dosages or to avoid side effects (11%). Finances (3.5%) or an inability to obtain desired AAS (6.5%) were not factors for most. An additional 1.6% indicated that alterations to their cycle stemmed from work and personal life-related issues or injury. Of those (5%) not planning their cycles, most determined their use based on body response and goals.

#### Injection practices

Injection has been noted as the most common method of self-administration of NMAAS [24,52] and our data showed this as well; a vast majority injected AAS (95%). Sharing of needles or multi-use vials was denied by an overwhelming majority (99%); a finding also consistent with other reviews [53-55]. Reusing of needles was rare (0.7%) and most (73%) used a clean needle to draw the solution into the syringe and a separate needle to inject. Infections resulting from injection were rare (7%).

Injectable AAS were preferred over oral compounds by most (77%), with health reasons and the belief of better results in comparison to oral AAS considered important. To a lesser extent, the ability to maintain a stable blood level was somewhat important, while ease of use, how the AAS made the individual feel, and the inability to obtain injectable AAS were of lesser importance.

#### Medical supervision of NMAAS

Most (66%) expressed a willingness to seek medical supervision and the preponderance (61%) obtained blood work at least once per year to assess the effects of NMAAS use on their physical health. However, NMAAS users often mistrust physicians and consider them uniformed regarding NMAAS [56]. Accordingly, more than half (58%) lacked sufficient trust in their physician to report their NMAAS use; 92% felt the medical community's knowledge about NMAAS use was lacking. In addition, almost all (99%) felt that the public has an inflamed view of NMAAS side effects.

## Discussion

### Who is using AAS?

NMAAS is largely an adult phenomenon; the median user was twenty-nine years old, agreeing with earlier reports [25,32]. Users were typically unmarried Caucasians in their 20s and 30s who initiated NMAAS use after reaching the age of majority. They were not active in organized sports. They were highly educated, gainfully employed, white collar workers earning an above average income; such high levels of functioning in terms of education, income, and employment are consistent findings [9,25] and are inconsistent with the popular view of substance abusers. In total, our findings belie the images of AAS users as mostly risk-taking teenagers, cheating athletes, and a group akin to traditional drug abusers.

One possible limitation is our use of the Internet and the potential bias toward a higher-functioning group. However, the similarities of this sample with others employing different methodologies [25,32,53] minimizes this concern. Because the Internet is now a primary source for both purchasing AAS [31] and NMAAS information [32], a wide range of users are likely familiar and comfortable with its use. Hence, our sample likely represents the non-elite athlete, adult NMAAS using population. Further, the use of the Internet controlled for potential geographical variation in NMAAS prevalence and related behaviors [53,57,58]. Finally, the Internet facilitated access to a large sample – the largest, to our knowledge, ever collected.

NMAAS use was rarely associated with athletics; most users did not compete in sports of any kind. In fact, relatively few had participated in high school sport and few reported using AAS at that time in their life. Contrary to portrayals of coaches and athletes as the primary consumers of AAS, only eight respondents were athletes or coaches by occupation; the results in this large sample agreed with those using smaller samples [25,32,52,59]; recreational weightlifters comprised almost 90% of our sample, also similar to reports from other reviewers [24]. NMAAS may, indeed, be prevalent among elite athletes, but competitive athletes are few among NMAAS users. Cheating in sport is a rare motivation for NMAAS and the small number of professional athletes using AAS generally competed in power sport events (e.g., power lifting, wrestling, football, full contact fighting). Interestingly, NMAAS was also reported in unexpected professional sports, such as rodeo, dance and tennis.

Bias must also be considered as a possible cause for low prevalence of athletes in our sample. The extent to which athletes use the Internet, both in general and as a source for AAS or for NMAAS information or read bodybuilding magazines is unknown. Competitive athletes may be less likely to volunteer to participate and provide such sensitive information. Conversely, as noted previously, the observed consistency between our findings and those from smaller datasets [59] suggests we have tapped the same population and we would expect that with the Internet serving as the primary source of AAS trade, athletes should be represented.

The largest yet least visible group of NMAAS users is recreational weightlifters with more varied reasons for use than competitive athletics [51,60]; "...a great deal of anabolic steroid use occurs in private gymnasia (non-local authority) among non-competitive recreational athletes [51]" and "...noncompetitive recreational users make up a large portion of the AAS-using population [25]." Our findings agree with this ubiquitous observation [10,25,32,51,58,60].

### What is being used?

Injectable AAS were most popular and preferred, due largely to decreased liver toxicity as compared to oral agents. Almost 10% exclusively injected AAS, having never used oral agents. Contrary to traditional notions that injection reflects escalation in drug use, intra-muscular (IM) injection of AAS avoids several of the more serious potential side effects of NMAAS and may be a less risky approach. Oral AAS are associated with liver damage [59,61] and IM injection of AAS "...could therefore be considered a rational attempt to reduce harm rather than an element of escalating use [9]" and may be "...more advisable... [62]." The prevalence and preference of injecting AAS suggests that injection should be considered the normative route of administration; a positive finding, in a public health sense, due to its potential reduction of harm.

Despite having reduced hepatotoxicity, intramuscular injection is not without potential complications; a small minority reported injection-site infection. Still, unlike other groups of illicit drug users [63-65], sharing of needles and multi-use vials, and reuse of needles were almost non-existent. The use of separate needles to draw and inject oil-based products was the standard approach. NMAAS users in general seemed to practice safe injection techniques [51,66] and NMAAS use apparently "...present [s] little risk of HIV transmission" [66] or other blood borne pathogens [53]. Accordingly, viral hepatitis and HIV infection were not reported by anyone in our sample.

### Why are AAS being used?

Sports and competitive bodybuilding did not motivate NMAAS use in this group. Amateur sports, bodybuilding and power lifting were rarely cited as motivators. Consistent with this, few acknowledged a fear of losing athletic abilities if they ceased AAS use.

The primary motivations for NMAAS were increased muscle mass, strength and physical attractiveness. Loss of muscle and strength were important concerns should access to NMAAS cease. Negative reinforcement (avoidance motivation) was not as important as positive reinforcement (anticipated gains) in NMAAS; positive effects were endorsed more frequently and highly than were concerns about avoiding negative effects upon cessation. Overall, cessation of AAS use was not a concern for many users. Although low self-esteem certainly may motivate some AAS users, it was not a primary motivator. In fact, loss of respect was the least endorsed fear. The most parsimonious explanation seems to be that NMAAS respondents, like most people, have an idea of how they wish to appear and, as a goal-directed group, adopted a structured NMAAS regimen, along with diet, exercise and other supportive components to attain a desired physique or outcome.

NMAAS appeared to be more associated with an image of the ideal (attractive) body structure and ability as large, muscular and powerful, a view that is consistent with Western ideals, and not with an aversion towards being small. Positive changes in strength and muscularity were more highly endorsed than were avoidance of loss of these characteristics. This is a subtle but important distinction; it suggests a desire to enhance one's physique, even when it leads to use of NMAAS, as motivation, as opposed to body dissatisfaction as psychopathology which leads to AAS use [67]. It is clear, however, that we did not measure satisfaction or dissatisfaction with current physique on our sample. Nonetheless, it has been noted that "...people actively use body image to achieve certain ends, justify particular actions and manage particular identities [68]" and AAS-using and non-using gym goers have comparable concerns about body image [69]. Hence, in goal-oriented NMAAS users, the desire for an improved physique may not reflect dissatisfaction with one's current physique but part of a strategy aimed at self-improvement and achieving their goals. Interestingly, even though increases in body esteem associated with NMAAS allegedly remitted after cessation of use [70], becoming less attractive upon cessation did not concern this group.

The top three motivators among this sample replicated those in two Australian surveys [i.e., [25,71]]. Wright and colleagues (2001) [62] also found increased muscle mass as the primary motivating factor. The use of AAS for fitness-related and cosmetic purposes is widely reported [7,8,24,47,71-74] and NMAAS use has been discussed as a form of appearance enhancement similar to plastic surgery [75]. Our data adds to a literature that suggests that users may consider NMAAS use as a means to enhance normal functioning, which is a growing trend in our society [76].

Motivations for use were generally stable across age groups, consistent with the observation by Brower, Elipulos, Blow, Catlin, & Beresford [27], (1990) that "...older and younger subjects did not appear to differ." It might have been expected that motivations for use would change with development, given the changing nature of roles across the lifespan. The minor differences that did appear primarily were associated with typical age-related biological changes (e.g., motivations for increasing endurance, decreasing fat); however, they may also reflect psychosocial development (e.g., attracting sexual partners, increases in confidence). In any case, although statistically significant, the magnitude of these age-related changes was less than might be expected.

It has been suggested [77] that many AAS users experience a "high" from use, although others [78] found such reports to be rare. Our results agree with the latter notion; the great preponderance of our respondents (99%) denied that immediate psychogenic effects (e.g., intoxication, arousal or euphoria) motivated their use, dose, duration or frequency of use, suggesting that they did not experience AAS as euphorigenic [6,72] and did not inject for a "high."

### When are AAS being used?

Initiation of NMAAS use was an adult phenomenon; onset occurred in the great majority (94%) after reaching eighteen years of age and only 6% of current users initiated NMAAS prior to that age. Reports of age of onset in the literature vary; our results agree with some reports [21] but not others [79]. It appears, however, that the typical adult male American using AAS initiated NMAAS in his mid-twenties [see also [24,25]], within 5 years of beginning weight training. This does not minimize concerns about adolescent NMAAS; significant numbers of adolescents are experimenting with AAS (although surveys suggest that many more experiment with and use other drugs). But adolescent onset of use was rare among ongoing adult users, suggesting a discontinuity between adult NMAAS and adolescent experimentation. Adolescent experimentation may be qualitatively different than adult use, given the developmental issues involved in adolescent drug use/experimentation, and may not invariably lead to longer-term use. Of course, the best data to explore this issue would come from true longitudinal studies as opposed to retrospective reports of onset. Nonetheless, given the potential negative effects of adolescent use, research efforts should focus on exploring adolescents' patterns of and motivations for NMAAS to more fully inform identification of those at risk and efforts to prevent use.

Ultimately, in the absence of longitudinal studies [80], it is impossible to make definitive statements about the relationship between patterns of initiation and long-term use. It is noteworthy that the prevalence of adult onset we observed differs from the pattern of initiation seen in other drugs [e.g., alcohol; [81]] where early onset predicts later use. However, research has shown clear distinction between AAS users and those using other generally illicit drugs [82].

### How are AAS being used?

The overall fitness and lifestyle context in which NMAAS is embedded is likely inconsistent with widespread use; as Korkia [58] (1994) noted, few "...are prepared to take regular and vigorous exercise like weight-training, which must accompany AS use, and therefore it is unlikely that AS use would reach epidemic proportions." This is the context of NMAAS; the majority of users maintained a strenuous regular training regimen, lifting weights 4–5 days per week, as well as a strict dietary regimen high in protein and low in fats and sugars.

AAS were used about six months per year, broken up into 3 month periods, reflecting common cycling practices employed to allow the body to return to homeostasis. Periods of use were largely planned in great detail and the necessary drugs were most often in hand ahead of time. Ancillary drugs – drugs used to prevent or treat AAS related side effects or make AAS more effective – were relatively commonplace. NMAAS users utilize SERMs (i.e., clomid [clomiphene citrate], nolvadex [tamoxifen citrate] which block estrogen receptors) or aromatase inhibitors (i.e., arimidex [anastrozole] which block the conversion of AAS into estrogen) because in an attempt to maintain homeostasis, the body converts excess androgens into estrogen, resulting in unwanted side effects. The use of peptides (i.e., HGH, IGF-1, insulin) has received little attention in the realm of NMAAS users; however the availability of recumbent forms of peptides has lead to greater use of these hormones by non-athletes [83]. HGH, although taken with AAS, is often combined with insulin or thyroid hormones (t3/t4). Insulin, familiar to many only as a medication used in the treatment of diabetes, is a very anabolic compound that shuttles needed nutrients to muscles, produces growth factors when combined with HGH in the liver and combats insulin resistance produced by HGH. Thyroid hormones burn fat and NMAAS users may combine them with HGH to increase their levels which is reduced by HGH.

This data raises two interesting points. First, NMAAS involves more forethought and organization than other illicit drug use; it is less impulsive and more considered. The planned cycling, healthy diet, ancillary drugs, blood work, and mitigation of harm via route of administration suggest a strategic approach meant to maximize benefits and minimize harm. Second, pre-planning required users to obtain most of their planned cycle prior to beginning. Hence, unlike other illicit drugs procured by end-users in single or short-term use quantities, AAS users are likely to have substantial amounts of AAS on hand for long-term personal use. To achieve supraphysiological levels of steroid hormones, many respondents used up to 12 methandrostenolone tablets (5 mg each) per day, with a few using over 20 tablets. This reasonably necessitates an initial possession of 1,000 tablets or more for personal use (consistent with anecdotal observations of AAS purchasing patterns; [84]). Such quantities, in the case of single-use illicit drugs, would suggest intent to distribute; in NMAAS they are more likely an on-hand quantity for personal use. The legal implications of this are that some AAS users may be improperly accused of trafficking based solely upon the quantity recovered.

AAS users are well known for being educated on the drugs they use and most seek information about AAS at least monthly [25]. Most recognized the value of medical supervision and regular blood work, but did not trust their physician enough to inform them of their NMAAS. Consistent with other studies [56,69], they almost universally lacked confidence in physicians knowledge of AAS; a sentiment with which physicians seem to agree [60]. As a result, NMAAS users seek information from various non-medical sources [62].

## Conclusion

The picture of NMAAS use reported herein confirms and extends much of what previous research has shown about this subject. It differs from the common impression held by the media and public. High-functioning NMAAS users of approximately 30 years of age who do not compete athletically receive little attention in the larger discussion of NMAAS use and also bear little resemblance to the illicit drug abuser to whom they are often compared. These findings suggest that one size does not fit all.

These results suggest that most attempts to address NMAAS use have been off-target. NMAAS use emerged from the community of elite athletes, but it spread to non-athletes, where it is now more prevalent. The targeting of athletes through drug testing and other interventions does little to address use among non-competitive users. Additionally, condemnations of NMAAS use based on misuse by adolescents, even when it is purportedly associated with tragic deaths, do little to address use among the vast majority of users; they are not adolescents.

Attempts to devalue the accomplishments of sports figures accused of NMAAS are fraught with unintended consequences; communicating social and moral admonishment of "cheating" as a means to curtail use also highlights what may be seen as otherwise unattainable achievements, thus perhaps perpetuating use. We found NMAAS users to be a driven and ambitious group dedicated to gym attendance, diet, occupational and educational attainment. They view AAS as a form of enhancement that, when approached in an informed fashion is seen to have an acceptable cost/benefit ratio. They do not simply self-administer AAS and expect positive effects or achieve goals; most use AAS in conjunction with considerable effort, including strict diet and workout regimens. The vast bulk of AAS users are not athletes and hence, are not likely to view themselves as cheaters, but rather as individuals using directed drug technology as one part of a strategy for physical self-improvement. In fact, this perception parallels current social trends; the use of medications and medical technology for enhancement is a growing phenomenon in our society [76].

A seeming contradiction runs through our data. In spite of possible limitations of the Internet for data collection, the segment of the population engaged in NMAAS that we accessed was an active, young, well-educated, and health-focused group. This health-centered lifestyle may seem clearly inconsistent with the potential complications of NMAAS. However, at least in the case of this sample, the use of AAS appeared well-considered; most attempt to use AAS *responsibly*, adopting what are perceived as safer routes of administration and hygienic injection practices, consuming a healthy diet, employing methods to reduce side effects, obtaining regular blood work, and periodically cycling on and off AAS.

Obviously none of this justifies NMAAS. But prevalence rates of NMAAS are at best stable, if not increasing, in spite of prevention programs, augmented law enforcement attention, increased legal penalties, state-mandated high school steroid testing programs, and various stricter sanctions by professional and amateur sports organizations. This disparity between levels of use and efforts to curtail it may largely reflect the virtually invisible nature of the largest segment of the AAS-using population: adult non-athletes. In contrast to current policies, several have called for harm reduction [60,62]. We, along with our colleagues [62], believe that if a harm reduction policy has merit, it must begin by regaining NMAAS users' trust. That process starts with looking beyond the conventional portrait of NMAAS to further explore how and why these drugs are used in the vast majority of users.

## Abbreviations

AAS – anabolic-androgenic steroid(s)

FSH – Follicle Stimulating Hormone

LH – Luteinizing Hormone

NMAAS – non-medical anabolic-androgenic steroid(s)

SERM – Selective Estrogen Receptor Modulator

## Competing interests

The author(s) declare that they have no competing interests.

## Authors' contributions

JC made contributions to the design, acquisition of data, analysis and interpretation of data and involved in drafting the manuscript and revising.

RC made contributions to the design, acquisition of data, analysis and interpretation of data and involved in drafting the manuscript and revising.

JD made contributions to the design, acquisition of data, analysis and interpretation of data and involved in drafting the manuscript and revising.

DG made contributions to the design, acquisition of data, analysis and interpretation of data and involved in drafting the manuscript and revising.

All authors read and approved the final manuscript.
